# Lipid-, Protein-, and Environmental Contamination Derived Off-Odor Volatile Compound Formation in Refrigerated Atlantic Salmon (*Salmo salar*) Fillets and the Role of Rearing Conditions

**DOI:** 10.3390/foods15091558

**Published:** 2026-04-30

**Authors:** Manpreet Kaur, Md Zakir Hossain, Kevin J. Fisher, Sheryl Barringer

**Affiliations:** 1 Department of Food Science and Technology, The Ohio State University, Columbus, OH 43210, USA; kaur.333@osu.edu (M.K.); hossain.154@osu.edu (M.Z.H.); 2 School of Environment and Natural Resources, The Ohio State University, Columbus, OH 43210, USA; fisher.645@osu.edu

**Keywords:** Atlantic salmon, refrigerated storage, off-odor volatiles, off-flavor, lipid oxidation, protein degradation, environmental contaminants, rearing conditions, SIFT-MS, shelf life

## Abstract

Atlantic salmon (*Salmo salar*) is highly perishable during refrigerated storage due to the formation of off-odor volatile compounds that limit shelf life and consumer acceptance. This study investigated the development of off-odor volatiles in Atlantic salmon fillets during refrigerated storage and evaluated how rearing conditions influence storage-induced volatile formation. Salmon reared under warm (20.3 ± 1.95 °C with continuous light) or cool (13.1 ± 0.85 °C with a 12 h light–12 h dark cycle) conditions were harvested, stored at 4 ± 1 °C, and analyzed at 0, 3, 7, 9, and 15 days using selected-ion flow-tube mass spectrometry (SIFT-MS). Refrigerated storage was the primary driver of volatile formation, with lipid-derived aldehydes and alcohols forming early, followed by additional oxidation products as deterioration progressed, and finally, terminal oxidation products. These findings demonstrate distinct temporal pathways of off-odor volatile formation during refrigerated storage, linking early-stage oxidation of polar lipids, mid-stage involvement of neutral lipids, and late-stage accumulation of terminal and microbial products. Protein-derived volatiles exhibited compound-specific behavior, with reactive sulfur- and nitrogen-containing compounds increasing early or mid-storage and microbial metabolites accumulating steadily over time. Environmentally derived off-odor compounds, including geosmin and 2-methylisoborneol, were progressively released during storage as lipid structures degraded. Warm-reared salmon consistently exhibited higher concentrations of lipid- and protein-derived volatiles, indicating greater oxidative and proteolytic susceptibility. Rearing conditions modulate the extent but not the progression of these spoilage mechanisms. This mechanistic understanding provides a basis for targeted strategies to control off-odor volatile compound development and improve refrigerated shelf life and sensory quality of Atlantic salmon.

## 1. Introduction

Atlantic salmon (*Salmo salar*) is one of the most widely consumed finfish worldwide due to its high nutritional value, favorable fatty acid composition, and desirable sensory attributes [1]. Despite these advantages, salmon is highly perishable, particularly during refrigerated storage, where rapid quality deterioration limits shelf life and marketability [1]. The development of off-odor volatile compounds during storage is a primary driver of sensory rejection and represents a major challenge for the seafood industry [2,3,4].

Refrigerated storage induces a series of biochemical and microbial processes that contribute to quality deterioration in fish [1,2,3]. Among these, lipid oxidation is a major pathway in salmon because of their high content of long-chain polyunsaturated fatty acids (PUFAs), which are particularly susceptible to oxidative reactions [1,2,3]. Lipid oxidation during storage leads to the formation of a wide range of volatile compounds, including aldehydes, alcohols, ketones, acids, and hydrocarbons, which are highly odor-active and associated with off-flavors [2,5,6]. These volatiles form through oxidation reactions involving lipid hydroperoxides and their subsequent decomposition products [2,5,6]. The composition and abundance of lipid-derived volatiles formed during storage depend on multiple factors, including fatty-acid composition, oxygen availability, presence of pro-oxidative catalysts, and storage conditions [2,5,6].

Protein degradation is another major contributor to off-odor formation during refrigerated storage. Endogenous proteolysis and microbial metabolism release amino acids and peptides that serve as precursors for nitrogen- and sulfur-containing volatile compounds, including amines, organic acids, alcohols, aldehydes, and sulfides [1]. Many of these compounds have extremely low odor thresholds and contribute disproportionately to sensory spoilage [1]. Importantly, lipid oxidation and protein degradation processes are closely linked. Oxidative damage to proteins can enhance proteolysis, while protein breakdown releases pro-oxidants, such as free iron and reactive peptides, that further accelerate lipid peroxidation [7,8,9]. These interactions complicate spoilage dynamics and lead to overlapping volatile formation pathways during storage.

In addition to volatiles formed post-harvest, salmon fillets also contain environmentally derived off-odor compounds that are accumulated prior to harvest [10,11,12]. Compounds such as geosmin and 2-methylisoborneol (2-MIB), produced by cyanobacteria and actinomycetes in aquaculture systems, are absorbed into lipid-rich tissues during rearing [10,11,12]. These compounds are not generated during refrigerated storage but can be released as lipid structures and muscle tissue deteriorate, contributing earthy, musty, or feed-related off-odors [13,14].

While refrigerated storage is the primary driver of volatile formation, pre-harvest rearing conditions can strongly influence how salmon muscle responds during storage. Rearing temperature affects growth rate, lipid deposition, membrane composition, antioxidant status, and overall redox balance in fish muscle, which can influence susceptibility to oxidation [15,16]. Salmon reared at higher temperatures have been shown to exhibit reduced endogenous antioxidant defenses, including lower levels of vitamin E, vitamin C, and glutathione [16,17,18], potentially predisposing the muscle to accelerated oxidative and proteolytic deterioration once refrigerated storage begins. However, the extent to which rearing temperature modifies storage-induced volatile formation across lipid-derived, protein-derived, and environmentally derived pathways remains insufficiently understood.

In our previous study [16], the effects of rearing, physiological, and processing conditions on the volatile profile of Atlantic salmon were investigated. Building on that work, the present study focuses on the development of volatile compounds during refrigerated storage, with particular emphasis on progression of lipid-, protein-, and environmentally derived off-odor pathways. A mechanistic understanding of how refrigerated storage initiates and drives volatile formation, and how rearing temperature influences these processes, is essential for improving seafood shelf-life prediction, quality control, and aquaculture production strategies. Therefore, the objective of this study was to first characterize changes in off-odor volatile compounds in Atlantic salmon fillets during refrigerated storage and then evaluate how rearing temperature influences these storage-induced volatile formation pathways. Volatile compounds derived from lipid oxidation, protein degradation, and environmental contamination were monitored over time to distinguish their behavior over time, biochemical origin, and contribution to spoilage odor development.

## 2. Materials and Methods

### 2.1. Experimental Design and Rearing Conditions

All animal handling and experimental procedures were approved by The Ohio State University Institutional Animal Care and Use Committee (IACUC protocol numbers 2008A0220-R5 and 2008A021-R5; approval date: 14 April 2024). Atlantic salmon were reared at the Aquaculture Laboratory, School of Environment and Natural Resources, The Ohio State University, using a recirculating aquaculture system (RAS) designed to allow controlled manipulation of water temperature and photoperiod. The RAS consisted of multiple 400 L tanks connected to a mechanical filtration unit and sump pump system. Water flow could be switched between recirculated chilled water and fresh municipal water using cutoff valves, allowing independent environmental control between tank groups. Fish density was adjusted throughout the growth period to support normal growth and welfare, with 33 fish per tank at the time of harvest. The rearing conditions, including temperature and photoperiod, have been described in detail previously [16] and were not independently varied in the present study. Briefly, fish were reared under two rearing conditions, and the fish analyzed in the present study were from the third harvest [16]. Fish were fed extruded salmon feed (Skretting, Tooele, UT, USA) ad libitum throughout the study.

### 2.2. Harvest and Sample Preparation

At the final harvest point, six female Atlantic salmon were randomly selected, consisting of three fish reared under warm conditions (20.3 ± 1.95 °C with continuous light) and three fish reared under cool conditions (13.1 ± 0.85 °C with a 12 h light–12 h dark cycle). Fish were humanely euthanized by mechanical stunning followed by immediate bleeding via dorsal artery severance. Each fish was filleted. The skin was removed manually to ensure complete separation from the muscle tissue. Only the muscle portion was used for subsequent analysis, with subcutaneous fat and red muscle largely excluded. Prepared samples were vacuum sealed and stored at −80 °C for up to 90 days prior to volatile analysis. Frozen storage at −80 °C was used to preserve the samples’ biochemical integrity and enable consistent, batch-wise analysis across all storage time points.

### 2.3. Refrigerated Storage and Sampling Timeline

Following frozen storage at −80 °C, muscle samples (skin-on and skin-off) were removed from the freezer and thawed at room temperature (22 ± 2 °C) for 30 min. After thawing, samples were immediately placed into food-grade polyethylene zip-lock bags and transferred to refrigeration at 4 ± 1 °C. Volatile analysis was performed immediately after thawing and prior to refrigerated storage to establish baseline values (day 0). After the initial analysis, samples were stored under refrigeration and subsequently analyzed after 3, 7, 9, and 15 days of refrigerated storage. Subsamples were repeatedly collected from the same individual fillet over the storage period at each time point. While this approach enabled tracking of volatile development within the same biological sample, it may introduce minor variability due to repeated exposure to oxygen, tissue disruption, and handling during subsampling. To minimize these effects, sample handling time was limited (approximately 2 min), and samples remained sealed between time points except during subsampling. All samples were stored on the same shelf within the refrigerator to ensure uniform temperature exposure throughout the storage period. Storage duration and sampling intervals were selected to capture the early, middle, and late stages of refrigerated storage-associated volatile development.

### 2.4. Headspace Volatile Analysis by SIFT-MS

A 2 g portion of minced muscle tissue from the head region of each fillet was transferred to a 500 mL Pyrex bottle. To facilitate volatile release, 20 mL of 0.5% (*v*/*v*) ethanol was added, and the bottles were sealed with open-top, septum-lined caps. Samples were homogenized using a vortex mixer for 1 min and equilibrated in water bath at 42 °C for 30 min. Headspace volatile compounds were analyzed using selected-ion flow-tube mass spectrometry (SIFT-MS; Voice200ultra, Syft Technologies, Christchurch, New Zealand). Analyses were performed in selected-ion monitoring mode using H_3_O^+^, NO^+^, and O_2_^+^ precursor ions. Volatile compounds quantified in this study were the same as in our previous study [16]. Compound concentrations were calculated using established ion–molecule reaction rate coefficients. Instrument calibration was verified using a certified gas standard containing benzene, ethylbenzene, toluene, and xylene isomers prior to sample analysis. Headspace sampling was conducted using a 14-gauge passivated needle, with the inlet temperature maintained at 175 °C. Each sample was analyzed over a 120 s acquisition period. Three analytical replicates were performed per sample type. An empty Pyrex bottle was used as a blank.

### 2.5. Fatty Acid Composition by GC

The lipids were extracted and separated into polar and lipid fractions [19]. Fatty acid composition was analyzed by GC using the method of [19].

### 2.6. Statistical Analysis

Statistical analyses were conducted using JMP^®^ Pro Version 16.0.0 (SAS Institute Inc., Cary, NC, USA). Figures were generated using MATLAB^®^ R2024b Update 5 (MathWorks, Natick, MA, USA). Two-way analysis of variance (ANOVA) was applied to assess the effects of rearing condition × refrigeration over time. Fisher’s Least Significant Difference (LSD) test was used for post hoc comparisons. Statistical significance was defined at *p* ≤ 0.05. For each factor, three biological replicates were included, with a total of six fish analyzed per harvest.

## 3. Results

Volatiles from raw Atlantic salmon fillets were tested over a period of 0, 3, 7, 9, and 15 days of refrigerated storage. Deterioration of salmon quality progressed during refrigerated storage, as indicated by the production of volatile off odors. While similar, the patterns for off-odor creation differed depending on whether the source was lipid oxidation, protein degradation or volatiles from environmental contamination. These three sources of off-odor volatiles were therefore examined separately to assess their individual contributions to volatile generation.

### 3.1. Lipid-Derived Volatile Compounds in Atlantic Salmon Fillets

Lipid oxidation volatiles are primarily aldehydes, alcohols, ketones and hydrocarbons, which are derived from lipid breakdown (Table A1). These can be grouped into three distinct groups based on their formation over time. The first group showed a rapid increase, with a maximum at day 3, followed by a gradual decrease through day 15. They are strongly associated with oxidation of the polar lipid fraction long chain, highly unsaturated polyunsaturated fatty acids (PUFAs) docosahexaenoic acid (C22:6n3) (DHA), eicosapentaenoic acid (C20:5n3) (EPA), and arachidonic acid (C20:4n6) (ARA). The second group peaked between days 7 and 9 before declining and are likely from oxidation of both the polar and neutral lipids especially the short-chain monounsaturated fatty acid (MUFA) oleic acid and PUFAs alpha linolenic acid (C18:3n3) (ALA) and linoleic acid (C18:2n6) (LA). The third group showed a continuous increase over the entire 15-day storage period due to breakdown of the lipid chains and formation of terminal lipid oxidation products.

#### 3.1.1. First Group—Volatiles Formed from the Polar Lipid Fraction

**Figure 1:**
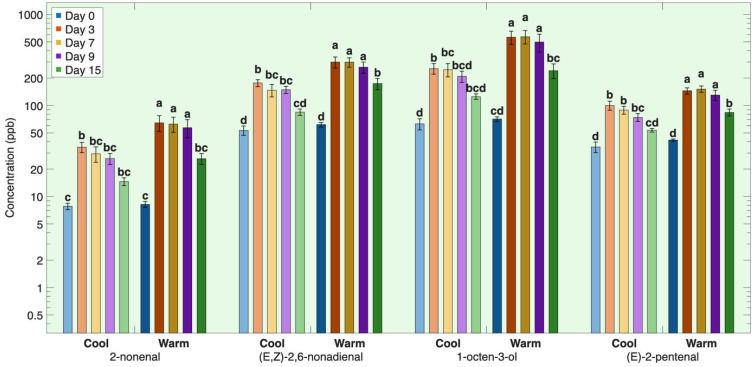
Group 1 lipid-derived volatiles over time during refrigeration for salmon reared under two different temperatures. Volatile concentration is plotted on a base-10 logarithmic (log_10_) scale. Different letters within the same volatile indicate significant differences among all storage times and rearing conditions (*p* < 0.05).

The first group consisted of lipid oxidation volatiles that exhibited a pronounced early increase in concentration, reaching peak concentrations by day 3, followed by a gradual decline through day 15 (Figure 1). This group included the volatile compounds 2-nonenal, (E,Z)-2,6-nonadienal, 1-octen-3-ol, oct-2-en-1-ol and (E)-2-pentenal (Table A1, Figure 1).

The first group comprises lipid oxidation products originating primarily from autooxidation of the polar lipid fraction’s highly unsaturated long-chain polyunsaturated fatty acids (PUFAs) [2,5,6]. Polar lipids are very susceptible to oxidative deterioration because membrane-associated fatty acids are directly exposed to pro-oxidants such as free iron, heme proteins, and endogenous enzymes released during postmortem muscle degradation during chilled storage [6,8,9,20,21,22]. The high degree of unsaturation of long-chain PUFAs concentrated within these polar lipid membranes accelerates autoxidation reactions, leading to the rapid formation of these lipid-derived volatile compounds [2,5].

The aldehydes and alcohol 2-nonenal, (E,Z)-2,6-nonadienal, 1-octen-3-ol, and (E)-2-pentenal are well-known oxidation products of DHA (C22:6n3), EPA (C20:5n3), and ARA (C20:4n6) in fish muscle [23,24,25,26]. The polar lipid fraction has twice the percentage of these long-chain PUFAs DHA (C22:6n3), EPA (C20:5n3), and ARA (C20:4n6) compared to the neutral fraction (Table 1).

These volatile compounds form stable adducts with muscle proteins via amino and sulfhydryl groups [7,8] and undergo further oxidation or reduction to secondary products [2,5,6], leading to a rapid decrease after 3 days. Although oxidation of these PUFAs in the neutral, or storage, lipids likely also contribute to the formation of these volatiles, the pronounced early increase suggests that oxidation of the polar, membrane-based, lipids is the dominant source during the first few days of storage.

#### 3.1.2. Second Group—Volatiles Formed from Polar and Neutral Lipids

**Figure 2:**
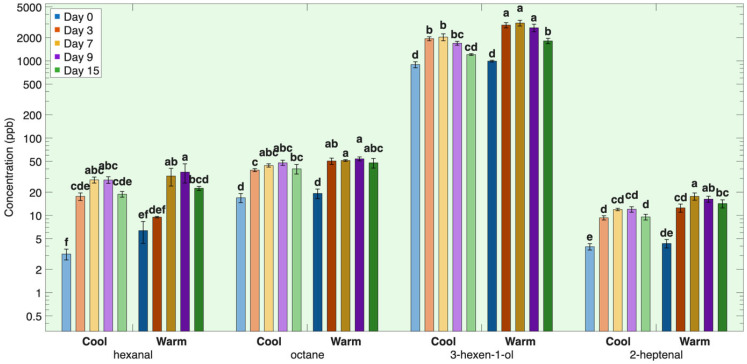
Group 2 lipid-derived volatiles over time during refrigeration for salmon reared under two different temperatures. Volatile concentration is plotted on a base-10 logarithmic (log_10_) scale. Different letters within the same volatile indicate significant differences among all storage times and rearing conditions (*p* < 0.05).

The second group of lipid-derived volatiles contains a larger number of volatiles that reached their highest concentrations around days 7–9 of storage and then declined (Figure 2). This group included the volatile compounds hexanal, 1-hexanol, 1-octanol, 2,4-heptadienal, 2-heptenal, 2-heptanone, 2,4-decadienal, 3-hexen-1-ol, heptanal, 2-undecanone, heptane, 1-octen-3-one, octane, propanal, 2-hexanal, 2-decenal, trans-2-undecenal, 2-nonanone, 2-decanone, 2-octene and nonanal (Table A1, Figure 2).

In contrast to the volatiles in the first group, which peaked on day 3, volatile compounds in the second group continued to increase through days 7–9, indicating ongoing lipid oxidation over time. The volatile compounds in this group mainly arise from lipid hydroperoxide breakdown of the monounsaturated fatty acid oleic acid and polyunsaturated fatty acids (PUFAs) linoleic acid and α-linolenic acid in neutral lipid depots.

The continuous increase in the concentration of volatile compounds in the second group through days 7–9 suggests an increasing contribution from neutral or storage lipids as storage progressed. The neutral lipid fractions are significantly higher in oleic acid (C18:1n9), linoleic acid (C18:2n6), and α-linolenic acid (C18:3n3) relative to the polar lipid fractions (Table 1). These are recognized precursors of the aldehydes, alcohols, ketones, and hydrocarbons in the second group. Specifically, oleic acid (C18:1n9) was predominantly associated with neutral lipids, accounting for approximately 32% of neutral lipids compared to 11–12% in polar lipids. Similarly, linoleic acid (C18:2n6) was 12–13% of neutral lipids compared to 4–5% in polar lipids and α-linolenic acid (C18:3n3) was 1% of the neutral lipid fraction as compared to 0.5% in polar lipids (Table 1).

In this group, volatile concentrations continued to increase until days 7–9 as oxidation extended beyond membrane-associated polar lipids and increasingly involved neutral, storage lipids. Neutral lipids, stored primarily as triacylglycerols within adipocytes or intracellular lipid droplets, are initially less accessible to oxidative reactions but contribute more strongly once oxidation progresses deeper into the lipid matrix [20,21,27,28,29].

The unsaturated aldehydes 2-heptenal, 2-decenal, trans-2-undecenal, 2,4-heptadienal, and 2,4-decadienal are well-established markers of MUFA and PUFA oxidation [30,31,32]. These compounds are characteristic products of continued n-6 PUFA oxidation, formed through hydroperoxide cleavage and subsequent β-scission reactions, and tend to persist once generated [30,31,32,33]. The primary fatty acid precursors for these aldehydes are linoleic acid (C18:2n-6), linolenic acid (C18:3n-3), and oleic acid (C18:1n-9) [30,31,32].

Saturated aldehydes such as hexanal, heptanal, propanal, nonanal, and 2-hexenal are typical secondary oxidation products formed from further decomposition of lipid hydroperoxides and generally increase through mid-storage as oxidation progresses [34,35]. Hexanal is primarily derived from oxidation of linoleic acid (C18:2n-6) and arachidonic acid (C20:4n-6) [31,32,36]. Heptanal is mainly associated with oxidation of linoleic acid (C18:2n-6) [30,31,32]. Propanal is formed from oxidation of n-3 polyunsaturated fatty acids, including α-linolenic acid (C18:3n-3), eicosapentaenoic acid (C20:5n-3; EPA), and docosahexaenoic acid (C22:6n-3; DHA) [37,38,39]. 2-Hexenal is derived primarily from linoleic acid (C18:2n-6), while nonanal originates mainly from oxidation of oleic acid (C18:1n-9) [40,41,42].

Alcohols including 1-hexanol, 3-hexen-1-ol, and 1-octanol are primarily formed via reduction of their corresponding aldehydes [43,44]. Their formation is associated mainly with oxidation of linoleic acid (C18:2n-6), α-linolenic acid (C18:3n-3), and oleic acid (C18:1n-9) [43,45].

Ketones such as 2-heptanone, 2-nonanone, 2-decanone, 2-undecanone, and 1-octen-3-one arise from peroxy radicals via biomolecular termination reactions and via oxidation of alcohol intermediates formed by alkoxy radicals [46,47]. These compounds form from degradation of unsaturated aldehydes and alcohols originating primarily from oxidation of the PUFAs linoleic acid (C18:2n-6), α-linolenic acid (C18:3n-3), arachidonic acid (C20:4n-6), EPA (C20:5n-3), and DHA (C22:6n-3) [41,45,48,49,50].

Hydrocarbons including heptane, octane, and 2-octene represent advanced oxidation markers formed through fatty acid fragmentation and dehydration reactions, primarily from oleic acid (C18:1n-9) and linoleic acid (C18:2n-6) [51,52].

After reaching maximum concentrations around days 7–9, these volatiles declined due to secondary reactions. Aldehydes and ketones can also bind with muscle proteins via amino and sulfhydryl groups [7,8], while others undergo further oxidation or reduction to alcohols, acids, or additional secondary metabolites [2,5,6].

#### 3.1.3. Third Group—Terminal Lipid Oxidation Products

**Figure 3:**
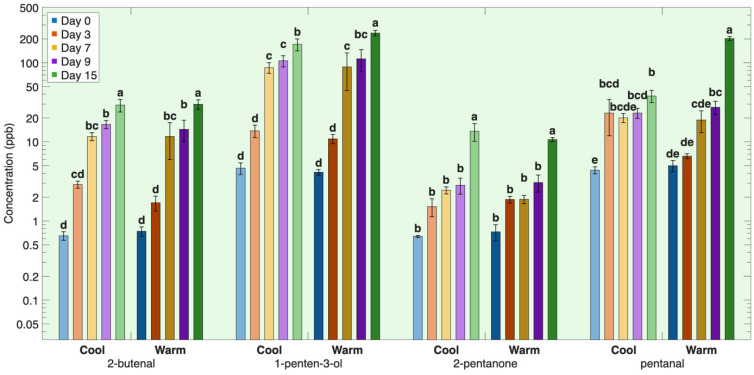
Group 3 lipid-derived volatiles over time during refrigeration for salmon reared under two different temperatures. Volatile concentration is plotted on a base-10 logarithmic (log_10_) scale. Different letters indicate significant differences within the same volatile among all storage times and rearing conditions (*p* < 0.05).

The third group consisted of volatile compounds that continued to increase throughout the full 15-day storage period. These included the volatile compounds 2-butenal, 1-penten-3-ol, 2-pentanone, hexanoic acid, pentanal, decanal, octanal, 1-pentanol, 2-octenal, 3-hexenal, 2-penten-1-ol, 2-pentene, and pentane (Table A1, Figure 3).

This group of volatiles is formed from slower lipid degradation and secondary transformation processes. The aldehydes pentanal, decanal, octanal, and 2-octenal arise from cleavage of fatty acid hydroperoxides mainly derived from oleic acid and linoleic acids, and from further transformation of longer-chain aldehydes via alkoxy-radical β-scission, generated earlier during lipid oxidation [52,53]. The sustained formation of these aldehydes indicates ongoing breakdown of lipid acyl chains as opposed to the rapid oxidation of membrane-based PUFAs [52,53,54]. Shorter-chain carbonyl compounds, such as 2-butenal and 2-pentanone, are formed from progressive chain shortening of oxidized fatty acids, including β-oxidation-related reactions and secondary scission of lipid-derived intermediates, which become more prominent as oxidation proceeds [2,5,55]. The alcohols 1-pentanol, 1-penten-3-ol, and 2-penten-1-ol are formed primarily through reduction of corresponding aldehydes, yielding more chemically stable products that accumulate once formed [49,56]. Similarly, hexanoic acid is formed by further oxidation of aldehydes, such as hexanal or alcohols, to carboxylic acids during extended storage [6,57,58]. The hydrocarbons pentane and 2-pentene are terminal lipid degradation products generated through radical-mediated fragmentation of lipid intermediates during secondary autoxidation reactions [59].

The steady accumulation of these compounds throughout storage indicates that their rate of formation exceeds their rate of degradation, reflecting continued breakdown of both polar and neutral lipid fractions as oxidation progresses over time.

#### 3.1.4. Effects of Rearing Conditions on Lipid-Derived Volatile Compounds During Refrigerated Storage

Atlantic salmon were reared under two different conditions—cool-rearing condition (13.1 ± 0.85 °C) with a 12 h light–12 h dark photoperiod, and a warm-rearing condition (20.3 ± 1.95 °C) with continuous light (24 h). These rearing environments influence fish metabolism, lipid deposition, oxidative stress, microbial exposure, and contaminant accumulation. The refrigerated fillets of salmon reared in warm conditions consistently showed higher concentrations of lipid-derived volatiles than the fillets of salmon reared in cold conditions (Table A1, Figure 1 and Figure 2). This indicates that rearing conditions significantly affected the rate of lipid oxidation in stored fillets. When the fish is alive, cool rearing conditions are ideal for Atlantic salmon, whereas warm conditions induce oxidative stress [15,16] that persists after the fillet is harvested. This increased lipid oxidation has been attributed to a combination of redox imbalance and increased substrate availability under warm rearing conditions [15,16].

Higher rearing temperatures and 24 h light conditions have been shown to reduce tissue levels of endogenous antioxidants such as vitamin C, vitamin E, and glutathione (GSH), shifting muscle toward a more oxidized redox state [16,17,18]. Such conditions favor faster lipid peroxidation, diminished thiol-based protection, and suppression of glutathione-cycle enzymes [60,61,62,63]. Consistent with this mechanism, warm-reared salmon in the present study exhibited earlier and higher concentrations of lipid-oxidation derived volatiles, indicating that reduced antioxidant capacity under elevated rearing temperatures accelerates oxidative reactions during storage.

Rearing temperature had a strong influence on the concentrations of both the first and second group volatiles, indicating a common underlying mechanism linked to enhanced oxidative susceptibility. There were no significant differences between warm- and cold-reared salmon on day 0, but warm-reared fish showed significantly higher concentrations of all first group volatiles from day 3 onward, while group 2 volatiles tended to show significantly higher concentrations in warm-reared fish around days 7–9. These included aldehydes (trans-2-undecenal, 2-heptenal, 2,4-decadienal, heptanal, nonanal), alcohols (oct-2-en-1-ol, 3-hexen-1-ol, 1-octanol), ketones (2-heptanone), and hydrocarbons (2-octene, octane). This increase in concentration over time indicates that the rearing conditions primarily affect oxidative processes during storage rather than volatile concentration while the fish is alive.

This effect can be attributed to oxidation of fatty acids that accumulated in Atlantic salmon during storage as a result of rearing conditions. Fish reared in warm rearing conditions had significantly higher concentrations of oleic acid, alpha linolenic acid, and linoleic acid than fish reared in cool temperatures (Table 1). These highly unsaturated fatty acids are rapidly oxidized and serve as key precursors of the volatile compounds (Table A1, Figure 1 and Figure 2).

Rearing temperature showed no effect on volatile compounds in group 3, suggesting that rearing temperature has no effect on the late-stage accumulation of these end-product volatiles (Table A1, Figure 3). This behavior is consistent with late-stage degradation processes that proceed in both groups once oxidation becomes established, reducing the relative impact of initial differences in lipid composition or oxidative status.

### 3.2. Protein-Derived Volatile Compounds in Atlantic Salmon Fillet

Protein-derived volatile compounds in Atlantic salmon fillets formed during refrigerated storage because of progressive proteolysis and subsequent microbial metabolism of released nitrogenous and sulfur-containing substrates (Table A2). These volatiles can be separated into two distinct groups, reflecting differences in chemical reactivity, precursor availability, and microbial involvement. The first group consists of volatiles that increased, peaked, and declined, and the second group comprises volatiles that increased continuously throughout refrigerated storage.

**Figure 4:**
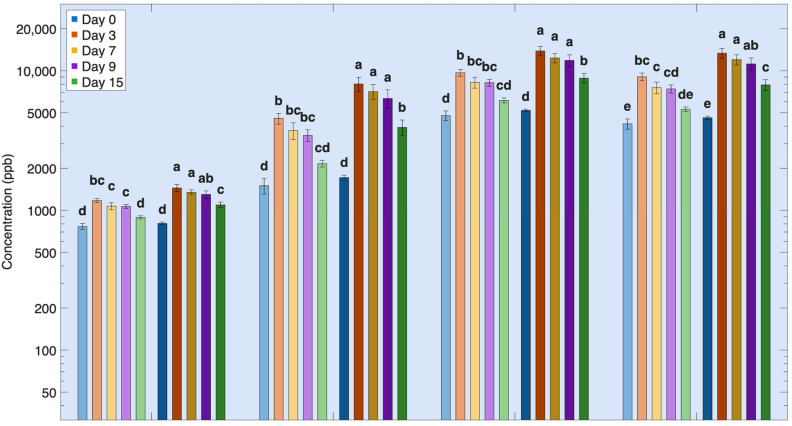
First Group protein degradation volatile compounds over time during refrigeration for salmon reared under two different temperatures. Volatile concentration is plotted on a base-10 logarithmic (log_10_) scale. Different letters indicate significant differences within the same volatile among all storage times and rearing conditions (*p* < 0.05).

The first group, where reactive protein-derived volatiles increased, peaked, and declined, included formaldehyde, carbon disulfide, indole, dimethyl disulfide, methyl mercaptan, 3-methyl-1-butanol, and isobutyl alcohol (Table A2, Figure 4). These compounds are formed relatively early during storage through amino acid degradation pathways via microbial catabolism or oxidation/interconversion pathways. Formaldehyde is generated through enzymatic action on trimethylamine oxide (TMAO) and oxidative cleavage of amino acid side chains [64,65,66] but is highly reactive and readily undergoes secondary reactions with amino groups in proteins and peptides, leading to a decline in headspace concentration despite continued protein degradation [67,68]. Carbon disulfide and methyl mercaptan originate from sulfur-containing amino acids, particularly methionine and cysteine, while dimethyl disulfide forms through oxidation and interconversion of methanethiol intermediates [69,70,71]. Indole arises from microbial catabolism of tryptophan or from feed [72,73] and branched-chain alcohols, such as 3-methyl-1-butanol and isobutyl alcohol, are produced by microbial spoilage via deamination and decarboxylation pathways from leucine and valine, respectively [74,75,76]. The concentration of these compounds over time reflects a balance between formation and destruction processes. While they are produced early during protein degradation, their net concentrations are subsequently reduced by interconversion among related sulfur species, microbial succession, depletion of specific amino acid precursors, and reactions with exposed amino and sulfhydryl groups in muscle proteins as storage progresses. Therefore, their decline does not indicate cessation of protein degradation but rather increased consumption and transformation of these highly reactive volatile compounds.

**Figure 5:**
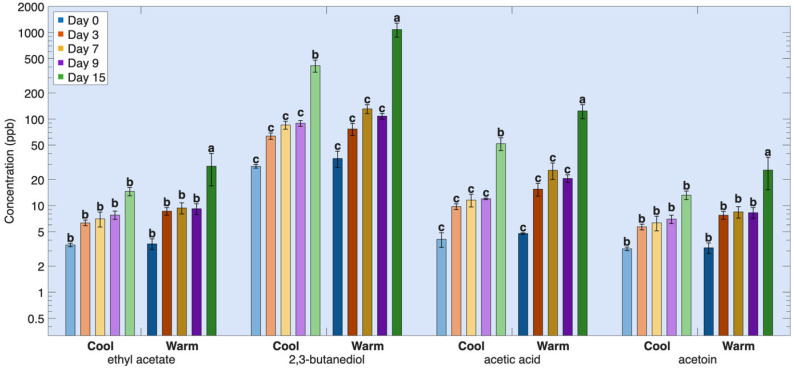
Second Group protein degradation volatile compounds over time during refrigeration for salmon reared under two different temperatures. Volatile concentration is plotted on a base-10 logarithmic (log_10_) scale. Different letters indicate significant differences within the same volatile among all storage times and rearing conditions (*p* < 0.05).

The second group, where protein-derived volatiles increased continuously throughout refrigerated storage, is attributed to sustained microbial action on the proteins, including proteolysis followed by amino acid catabolism and secondary reactions such as interconversion of intermediate metabolites formed during protein degradation and amino acid metabolism processes (Figure 5). This group includes dimethyl sulfide, trimethylamine, dimethylamine, ammonia, hydrogen sulfide, acetic acid, acetoin, 2,3-butanediol, ethyl acetate and acetone (Table A2, Figure 5).

These compounds are primarily formed through microbial pathways that remain active as storage advances and are less readily depleted because they are continuously replenished through ongoing proteolysis and amino acid metabolism, in which proteins are broken down into free amino acids that are subsequently converted into aldehydes, alcohols, acids, amines, and sulfur-containing volatiles via deamination, decarboxylation, and related microbial transformations. Trimethylamine increased steadily because of microbial reduction of trimethylamine oxide (TMAO), a naturally occurring osmolyte in marine fish [64,65,66]. Dimethylamine is formed from enzymatic action on trimethylamine [64,65,66]. Ammonia arose primarily from deamination during microbial metabolism of amino acids and peptides released by proteolysis [77,78]. Dimethyl sulfide and hydrogen sulfide originated from ongoing microbial degradation of sulfur-containing amino acids, with continued formation outweighing losses due to interconversion [79,80]. Acetic acid, acetoin, and 2,3-butanediol are secondary metabolites produced via mixed-acid and microbial pathways, often involving lipids or amino-acid derived intermediates [1,4,81,82], while ethyl acetate is formed as a secondary microbial ester through esterification of ethanol [83], with acetic acid generated during amino acid metabolism. Acetone represents a late-stage microbial metabolite associated with advanced spoilage [2]. Because these compounds are generated continuously and are not rapidly consumed through irreversible secondary reactions, their concentrations accumulated over time, reflecting the progressive nature of microbial spoilage during refrigerated storage.

#### Effects of Rearing Conditions on Protein-Derived Volatile Compounds During Refrigerated Storage

In general, the refrigerated fillets of Atlantic salmon reared in warm conditions exhibited higher volatile protein-derived concentrations than cool-reared salmon. For the first group, this was true for the entire storage period (days 3–15) but for the second group only on day 15, indicating that the susceptibility to protein degradation is accelerated under warm rearing conditions (Figure 4 and Figure 5, Table A2). Protein degradation pathways interact closely with lipid oxidation processes. Proteolysis and protein unfolding release pro-oxidants such as free iron and low-molecular-weight peptides, which accelerate lipid peroxidation [8,9]. Lipid oxidation promotes protein oxidation through aldehyde–protein interactions and iron-mediated oxidative reactions during storage [7]. Lipid-derived aldehydes react with exposed sulfhydryl and amine groups on muscle proteins, forming covalent adducts that remove aldehydes, such as hexanal and nonanal, from the headspace [7]. At the same time, other thiol- and amine-containing groups are converted into volatile sulfur and nitrogen compounds through microbial and endogenous enzymatic reactions. Enhanced lipid oxidation in warm-reared fish likely promotes higher protein oxidation through hemoglobin- and iron-mediated reactions, increasing the susceptibility of muscle proteins and their constituent amino acids to degradation and amplifying the formation of protein-derived volatiles (Figure 5) [69].

Trimethylamine (TMA) is widely recognized as a key protein-derived off-odor compound in fish and a common indicator of spoilage. It was the only protein-derived volatile not affected by the rearing temperature. Its formation is driven primarily by reduction of trimethylamine N-oxide (TMAO), a naturally occurring osmolyte present in fish muscle prior to harvest, so it showed weaker dependence on rearing temperature, consistent with its distinct formation pathway. The other volatiles in the second group were only significantly higher by the 15th day of storage.

### 3.3. Environmental Contamination-Derived Volatile Compounds in Atlantic Salmon Fillets

In addition to volatiles formed through lipid oxidation and protein degradation, Atlantic salmon fillets also contain environmentally derived off-odor compounds that accumulate prior to harvest and are released from the tissue during refrigerated storage (Table A3). Geosmin and 2-methylisoborneol (2-MIB) are well-known earthy/musty off-odor compounds commonly associated with fish and aquaculture products [10,11,12]. These compounds originate primarily from the fish rearing environment, where they are produced by cyanobacteria and actinomycetes [13,14]. These compounds are not formed during post-harvest refrigerated storage but accumulate in lipid-rich tissues of the fillet prior to harvest so their concentrations in the headspace increase as tissue structure deteriorates.

**Figure 6:**
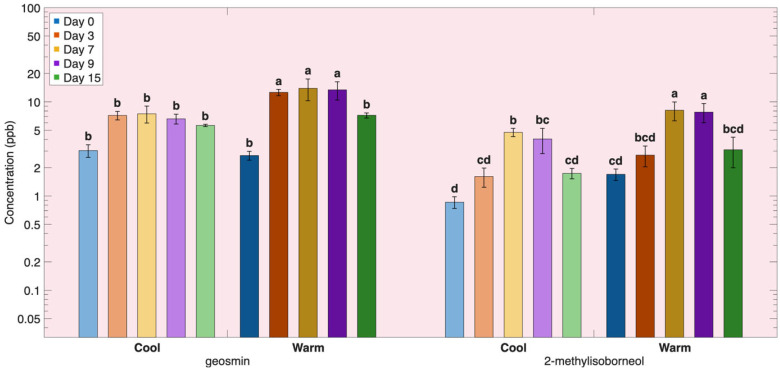
Environmental contamination-derived volatile compounds over time during refrigeration for salmon reared under two different temperatures. Volatile concentration is plotted on a base-10 logarithmic (log_10_) scale. Different letters within the same volatile indicate significant differences among all storage times and rearing conditions (*p* < 0.05).

During refrigerated storage, both geosmin and 2-MIB displayed patterns similar to lipid-derived degradation volatiles. Geosmin increased sharply at the beginning of storage, peaking by day 3, and then declined by day 15 (Table A3, Figure 6). This behavior suggests that geosmin is released as lipid oxidation advances. Geosmin closely resembles the group 1 lipid oxidation volatiles (Figure 1), suggesting a close relationship with polar membrane lipids. Geosmin is known to accumulate within polar membrane lipids [84], which are highly susceptible to oxidative damage and begin degrading early during refrigerated storage [8,9]. Oxidation of these membrane lipids leads to disruption of cellular integrity and facilitates the gradual release of hydrophobic compounds retained within the lipid bilayer. As oxidative damage and muscle tissue degradation progress, the geosmin accumulated in polar lipids is increasingly released into the headspace. This is consistent with previous studies showing that polar lipid oxidation initiates early during storage and plays a key role in the release of lipid-associated off-odor compounds [8,9,82].

2-Methylisoborneol (2-MIB) increased more gradually during refrigerated storage, reached a peak around day 7, and then declined toward day 15 (Table A3, Figure 6). 2-Methylisoborneol closely resembles the group 2 lipid oxidation volatiles (Figure 2), suggesting that the release of 2-MIB during storage is linked to the degradation of neutral lipid depots rather than membrane lipids. Neutral lipids oxidize more slowly. As neutral lipid oxidation progresses, disruption of lipid droplets facilitates the release of hydrophobic compounds retained within these lipid fractions [5], resulting in the day 7–9 storage peak observed for 2-MIB followed by decline.

#### Effects of Rearing Conditions on Environmental Contamination-Derived Volatile Compounds During Refrigerated Storage

Across all storage times, both geosmin and 2-methylisoborneol (2-MIB) were consistently higher in warm-reared salmon compared with cool-reared salmon (Table A3, Figure 6). Because these compounds are environmentally derived and are not formed during storage [12], differences observed during storage likely reflect differences in tissue oxidative status and structural integrity, which led to their release from muscle tissue. This suggests that rearing conditions influence the rate and extent of contaminant release from tissue during refrigerated storage. Warm-reared salmon exhibited greater lipid oxidation and tissue degradation during storage, which likely facilitated the progressive release of hydrophobic contaminants, such as geosmin and 2-MIB, that are physically partitioned within lipid membranes and neutral lipid depots [85,86]. As oxidative and structural breakdown of lipid fractions proceeds, these compounds become more readily released into the headspace. Cool-reared salmon displayed greater oxidative stability during storage, resulting in reduced lipid and membrane deterioration and consequently lower release of environmentally derived off-odor compounds. Rearing conditions influence the extent to which geosmin and 2-MIB are released during refrigerated storage, primarily by influencing lipid oxidation and tissue breakdown.

## 4. Conclusions

During refrigerated storage, off-odor development in Atlantic salmon fillets was caused by formation and release of volatile compounds derived from lipid oxidation, protein degradation, and pre-harvest environmental contamination. Lipid-derived volatiles exhibited distinct patterns that reflected the progression of oxidation from polar membrane phospholipids to neutral lipid fractions, with early-forming aldehydes and alcohols followed by secondary and tertiary oxidation products as storage advanced. Protein-derived volatiles also followed pathway-dependent trends, with highly reactive sulfur- and nitrogen-containing compounds showing early or mid-storage increases, while microbially derived metabolites, such as amines, organic acids, and fermentation products, accumulated steadily throughout storage. Environmentally derived off-odor compounds, such as geosmin and 2-methylisoborneol, were not formed during storage but were progressively released as lipid structures and muscle tissue deteriorated. Geosmin appears to deposit in the membrane lipids and 2-methylisoborneol in the storage lipids.

Rearing temperature significantly affected these storage-induced processes. Salmon reared under warmer conditions consistently exhibited higher concentrations of lipid- and protein-derived volatiles during refrigerated storage, indicating greater oxidative and proteolytic susceptibility. This effect is attributed to differences in lipid composition, relative polar lipid content, and reduced endogenous antioxidant protection prior to harvest. Enhanced lipid oxidation in warm-reared salmon further promoted protein degradation through iron-mediated and oxidative interactions, amplifying volatile formation across multiple pathways. Environmentally derived compounds were also released to a greater extent in warm-reared salmon, reflecting accelerated lipid and tissue degradation.

These findings demonstrate that spoilage odor development in refrigerated Atlantic salmon is governed by the interaction between post-harvest storage processes and pre-harvest rearing conditions. Refrigerated storage initiates and drives volatile formation, while rearing temperature determines the susceptibility of muscle tissue to oxidative and proteolytic deterioration. Understanding these interactions provides mechanistic insight into shelf-life variability in salmon and highlights the importance of rearing practices in controlling post-harvest quality and sensory stability.

## Figures and Tables

**Table 1:** Concentration of key fatty acids of the neutral and polar lipid fractions of warm and cold reared Atlantic salmon fish fillets. Different letters within the same fatty acid indicate significant differences in lipid fractions and rearing conditions (*p* < 0.05).

Fatty Acids	Polar—Warm (%)	Polar—Cool (%)	Neutral—Warm (%)	Neutral—Cool (%)
C22:6n3_docosahexaenoic acid	30.2 ^b^	34.1 ^a^	6.36 ^c^	6.62 ^c^
C20:5n3_eicosapentaenoic acid	6.94 ^a^	7.34 ^a^	2.58 ^b^	2.71 ^b^
C20:4n6_arachidonic acid	3.15 ^a^	3.38 ^a^	0.73 ^b^	0.72 ^b^
C18:1n9_oleic acid	12.6 ^b^	10.8 ^c^	31.8 ^a^	32.0 ^a^
C18:2n6_linoleic acid	5.03 ^c^	4.29 ^c^	13.37 ^a^	12.2 ^b^
C18:3n3_alpha linolenic acid	0.50 ^c^	0.46 ^d^	1.10 ^a^	1.03 ^b^

## Data Availability

The data presented in the study is available in Appendix A. Further inquiries can be directed to the corresponding author.

## Appendix A

**Table A1:** Lipid-derived off-odor volatile compounds (ppb) in fillets from Atlantic salmon reared at two different temperatures. The fillets were stored in the refrigerator for up to 15 days. Different letters within the same volatile indicate significant differences among all storage times and rearing conditions (*p* < 0.05).

Volatile (ppb)	Day 0 Cool	Day 3 Cool	Day 7 Cool	Day 9 Cool	Day 15 Cool	Day 0 Warm	Day 3 Warm	Day 7 Warm	Day 9 Warm	Day 15 Warm
First Group—Early-forming volatiles derived from polar lipid fraction										
2-nonenal	7.81 ^c^	34.8 ^b^	29.4 ^bc^	26.1 ^bc^	14.6 ^bc^	8.19 ^c^	64.4 ^a^	62.4 ^a^	57.0 ^a^	26.0 ^bc^
(E,Z)-2,6-nonadienal	53.2 ^d^	177 ^b^	147 ^bc^	149 ^bc^	84.6 ^cd^	61.6 ^d^	300 ^a^	299 ^a^	263 ^a^	174 ^b^
1-octen-3-ol	62.8 ^d^	254 ^b^	247 ^bc^	209 ^bcd^	125 ^bcd^	71.0 ^cd^	562 ^a^	569 ^a^	497 ^a^	241 ^bc^
oct-2-en-1-ol	54.4 ^d^	220 ^b^	213 ^bc^	181 ^bcd^	108 ^bcd^	61.5 ^cd^	486 ^a^	492 ^a^	430 ^a^	208 ^bc^
(E)-2-pentenal	35.0 ^d^	100 ^b^	89.0 ^b^	73.9 ^bc^	53.5 ^cd^	41.7 ^d^	145 ^a^	151 ^a^	130 ^a^	83.8 ^b^
Second Group—Mid-storage volatiles derived from polar and neutral lipid fractions										
hexanal	3.16 ^f^	17.6 ^cde^	28.8 ^abc^	28.7 ^abc^	18.8 ^cde^	6.34 ^ef^	9.47 ^def^	32.3 ^ab^	36.3 ^a^	22.4 ^bcd^
1-hexanol	1.58 ^e^	6.72 ^cd^	9.24 ^abc^	8.65 ^abc^	7.64 ^bc^	1.81 ^e^	4.02 ^de^	11.5 ^a^	10.6 ^ab^	7.45 ^c^
1-octanol	1.18 ^d^	4.39 ^bc^	6.58 ^ab^	6.65 ^ab^	5.53 ^abc^	1.59 ^d^	3.39 ^cd^	7.07 ^a^	5.16 ^abc^	6.14 ^ab^
2,4-heptadienal	12.7 ^b^	50.8 ^b^	230 ^a^	232 ^a^	44.0 ^b^	14.8 ^b^	28.7 ^b^	193 ^a^	196 ^a^	82.4 ^b^
2-heptenal	3.93 ^e^	9.30 ^d^	11.9 ^cd^	12.0 ^cd^	9.53 ^d^	4.32 ^de^	12.5 ^cd^	17.6 ^a^	16.2 ^ab^	14.2 ^bc^
2-heptanone	4.40 ^cde^	3.12 ^e^	5.66 ^bc^	4.98 ^bcd^	3.58 ^de^	4.84 ^bcde^	4.53 ^cde^	7.96 ^a^	6.44 ^ab^	5.75 ^bc^
2,4-decadienal	0.84 ^def^	0.61 ^f^	1.17 ^bcde^	0.95 ^cdef^	0.72 ^ef^	0.83 ^def^	1.32 ^abc^	1.65 ^a^	1.25 ^abcd^	1.44 ^ab^
3-hexen-1-ol	896 ^d^	1936 ^b^	2031 ^b^	1692 ^bc^	1206 ^cd^	992 ^d^	2901 ^a^	3082 ^a^	2677 ^a^	1813 ^b^
heptanal	1.87 ^e^	3.27 ^cd^	4.98 ^b^	4.14 ^bc^	3.60 ^c^	2.11 ^de^	4.16 ^bc^	7.19 ^a^	5.14 ^b^	5.32 ^b^
2-undecanone	0.66 ^a^	0.62 ^a^	0.77 ^a^	0.72 ^a^	0.73 ^a^	0.48 ^a^	1.06 ^a^	1.11 ^a^	0.69 ^a^	0.81 ^a^
heptane	54.4 ^e^	190 ^cde^	264 ^bc^	258 ^bcd^	217 ^bcde^	66.9 ^de^	247 ^bcde^	385 ^b^	410 ^b^	1158 ^a^
1-octen-3-one	0.36 ^b^	0.81 ^ab^	1.07 ^ab^	1.29 ^ab^	0.61 ^b^	0.81 ^ab^	0.76 ^ab^	1.67 ^a^	0.84 ^ab^	0.46 ^b^
octane	16.9 ^d^	38.5 ^c^	44.2 ^abc^	47.9 ^abc^	40.0 ^bc^	19.2 ^d^	50.5 ^ab^	51.3 ^a^	53.9 ^a^	47.9 ^abc^
propanal	8.52 ^b^	34.1 ^b^	155 ^a^	156 ^a^	29.5 ^b^	9.97 ^b^	19.3 ^b^	130 ^a^	132 ^a^	55.4 ^b^
2-hexenal	4.65 ^f^	13.1 ^cde^	12.8 ^cde^	13.6 ^bcd^	12.2 ^de^	6.95 ^ef^	15.0 ^abcd^	19.7 ^ab^	19.2 ^abc^	21.4 ^a^
2-decenal	0.96 ^c^	3.27 ^a^	2.62 ^ab^	1.61 ^bc^	2.38 ^ab^	1.57 ^bc^	2.71 ^ab^	2.99 ^a^	2.65 ^ab^	2.17 ^abc^
trans-2-undecenal	2.14 ^g^	7.03 ^bc^	4.43 ^def^	5.22 ^cde^	3.54 ^efg^	2.60 ^fg^	10.2 ^a^	10.6 ^a^	7.66 ^b^	5.72 ^bcd^
2-nonanone	0.69 ^b^	1.48 ^ab^	0.72 ^b^	0.96 ^ab^	1.48 ^ab^	0.61 ^b^	1.52 ^ab^	1.58 ^ab^	1.89 ^a^	1.25 ^ab^
2-decanone	0.51 ^b^	1.04 ^ab^	0.94 ^ab^	0.61 ^b^	0.56 ^b^	0.96 ^ab^	1.90 ^a^	1.80 ^a^	1.34 ^ab^	0.36 ^b^
2-octene	2.28 ^c^	5.93 ^abc^	4.64 ^bc^	4.72 ^bc^	4.91 ^bc^	2.44 ^c^	7.90 ^ab^	7.97 ^ab^	8.98 ^a^	6.94 ^ab^
nonanal	2.54 ^e^	9.76 ^bc^	9.58 ^bc^	7.21 ^cd^	5.26 ^de^	2.54 ^e^	15.3 ^a^	16.7 ^a^	13.2 ^ab^	8.71 ^cd^
Third Group—Terminal lipid oxidation products										
2-butenal	0.65 ^d^	2.89 ^cd^	11.7 ^bc^	16.6 ^b^	29.1 ^a^	0.74 ^d^	1.70 ^d^	11.7 ^bc^	14.4 ^b^	29.8 ^a^
1-penten-3-ol	4.65 ^d^	13.8 ^d^	86.8 ^c^	106 ^c^	171 ^b^	4.13 ^d^	10.9 ^d^	88.6 ^c^	112 ^bc^	237 ^a^
2-pentanone	0.64 ^b^	1.52 ^b^	2.45 ^b^	2.83 ^b^	13.6 ^a^	0.73 ^b^	1.87 ^b^	1.88 ^b^	3.06 ^b^	10.7 ^a^
hexanoic acid	0.57 ^d^	1.60 ^cd^	1.96 ^bc^	2.46 ^abc^	3.12 ^ab^	0.47 ^d^	1.63 ^cd^	3.50 ^a^	2.78 ^abc^	3.69 ^a^
pentanal	4.39 ^e^	23.1 ^bcd^	20.1 ^bcde^	23.1 ^bcd^	38.0 ^b^	4.99 ^de^	6.60 ^de^	18.9 ^cde^	27.3 ^bc^	202 ^a^
decanal	1.17 ^c^	1.90 ^bc^	1.81 ^bc^	1.54 ^bc^	2.02 ^bc^	1.00 ^c^	2.49 ^abc^	3.61 ^a^	2.14 ^abc^	2.90 ^ab^
octanal	1.25 ^d^	3.94 ^ab^	4.05 ^ab^	4.16 ^ab^	4.86 ^ab^	1.90 ^cd^	3.52 ^bc^	4.53 ^ab^	4.60 ^ab^	5.66 ^a^
1-pentanol	3.80 ^b^	10.2 ^b^	13.0 ^b^	12.8 ^b^	54.5 ^b^	4.20 ^b^	9.50 ^b^	15.7 ^b^	17.9 ^b^	311 ^a^
2-octenal	0.93 ^d^	1.50 ^bcd^	2.01 ^bcd^	1.97 ^bcd^	2.57 ^abc^	1.20 ^cd^	1.97 ^bcd^	3.49 ^a^	2.63 ^ab^	2.70 ^ab^
3-hexenal	1.75 ^a^	4.22 ^a^	2.53 ^a^	2.24 ^a^	4.47 ^a^	2.04 ^a^	4.98 ^a^	4.20 ^a^	4.29 ^a^	3.72 ^a^
2-penten-1-ol	3.43 ^d^	10.2 ^d^	64.0 ^bc^	78.0 ^c^	126 ^b^	3.05 ^d^	8.01 ^d^	65.3 ^c^	82.2 ^bc^	174 ^a^
2-pentene	5.60 ^b^	15.1 ^b^	19.3 ^b^	18.8 ^b^	80.5 ^b^	6.20 ^b^	14.0 ^b^	23.2 ^b^	26.4 ^b^	460 ^a^
pentane	1526 ^c^	2748 ^c^	3307 ^c^	3504 ^c^	17726 ^b^	1554 ^c^	2951 ^c^	4347 ^c^	3948 ^c^	41,929 ^a^

**Table A2:** Protein-derived off-odor volatile compounds (ppb) in warm and cold reared Atlantic salmon fillet. Different letters indicate significant differences within the same volatile among all storage times and rearing conditions (*p* < 0.05).

Volatile (ppb)	Day 0, Cool	Day 3, Cool	Day 7, Cool	Day 9, Cool	Day 15, Cool	Day 3, Warm	Day 7, Warm	Day 9, Warm	Day 15, Warm	Day 0, Warm
First Group										
formaldehyde	766 ^d^	1177 ^bc^	1071 ^c^	1067 ^c^	890 ^d^	1445 ^a^	1346 ^a^	1298 ^ab^	1094 ^c^	806 ^d^
carbon disulfide	329 ^d^	885 ^b^	748 ^bc^	712 ^bc^	432 ^cd^	1577 ^a^	1299 ^a^	1264 ^a^	784 ^b^	348 ^d^
indole	0.48 ^bc^	0.61 ^abc^	0.99 ^ab^	0.70 ^abc^	0.91 ^abc^	0.93 ^abc^	0.98 ^ab^	0.79 ^abc^	1.04 ^a^	0.45 ^c^
dimethyl disulfide	1500 ^d^	4548 ^b^	3729 ^bc^	3442 ^bc^	2160 ^cd^	8031 ^a^	7081 ^a^	6328 ^a^	3925 ^b^	1718 ^d^
methyl mercaptan	4772 ^d^	9671 ^b^	8248 ^bc^	8205 ^bc^	6128 ^cd^	13853 ^a^	12339 ^a^	11850 ^a^	8840 ^b^	5198 ^d^
3-methyl-1-butanol	12.5 ^e^	41.6 ^cde^	84.2 ^bc^	86.8 ^b^	60.6 ^bcd^	35.9 ^de^	100 ^b^	97.6 ^b^	175 ^a^	22.0 ^de^
isobutyl alcohol	4166 ^e^	9051 ^bc^	7575 ^c^	7411 ^cd^	5289 ^de^	13350 ^a^	12003 ^a^	11193 ^ab^	7921 ^c^	4606 ^e^
Second Group										
dimethyl sulfide	1.32 ^ef^	2.14 ^cde^	1.75 ^def^	1.94 ^cde^	2.83 ^bc^	3.20 ^ab^	2.76 ^bc^	2.62 ^bcd^	3.96 ^a^	1.02 ^f^
trimethylamine	22.6 ^c^	42.5 ^bc^	75.4 ^a^	84.0 ^a^	68.3 ^ab^	37.9 ^c^	75.3 ^a^	74.9 ^a^	72.8 ^a^	35.3 ^c^
dimethylamine	77.2 ^c^	274 ^b^	276 ^b^	289 ^b^	254 ^b^	511 ^a^	573 ^a^	528 ^a^	468 ^a^	84.1 ^c^
ammonia	375 ^cde^	438 ^bc^	344 ^e^	349 ^de^	449 ^bc^	496 ^ab^	420 ^bcde^	428 ^bcd^	559 ^a^	381 ^cde^
hydrogen sulfide	0.26 ^a^	0.58 ^a^	0.81 ^a^	1.71 ^a^	1.42 ^a^	0.39 ^a^	0.51 ^a^	0.50 ^a^	0.44 ^a^	0.43 ^a^
acetic acid	4.08 ^c^	9.76 ^c^	11.6 ^c^	12.0 ^c^	52.0 ^b^	15.5 ^c^	25.6 ^c^	20.6 ^c^	124 ^a^	4.76 ^c^
acetoin	3.17 ^b^	5.69 ^b^	6.33 ^b^	6.99 ^b^	13.2 ^b^	7.76 ^b^	8.47 ^b^	8.28 ^b^	25.7 ^a^	3.26 ^b^
2,3-butanediol	28.4 ^c^	63.7 ^c^	85.3 ^c^	89.3 ^c^	414 ^b^	76.7 ^c^	131 ^c^	108 ^c^	1081 ^a^	35.0 ^c^
ethyl acetate	3.52 ^b^	6.32 ^b^	7.04 ^b^	7.77 ^b^	14.6 ^b^	8.62 ^b^	9.41 ^b^	9.20 ^b^	28.5 ^a^	3.62 ^b^

**Table A3:** Environmental contamination-derived off-odor volatile compounds (ppb) in warm and cold reared Atlantic salmon fillet. Different letters indicate significant differences within the same volatile among all storage times and rearing conditions (*p* < 0.05).

Volatile (ppb)	Day 0, Cool	Day 3, Cool	Day 7, Cool	Day 9, Cool	Day 15, Cool	Day 0, Warm	Day 3, Warm	Day 7, Warm	Day 9, Warm	Day 15, Warm
geosmin	3.04 ^b^	7.17 ^b^	7.47 ^b^	6.60 ^b^	5.62 ^b^	2.69 ^b^	12.6 ^a^	13.9 ^a^	13.4 ^a^	7.20 ^b^
2-methylisoborneol	0.86 ^d^	1.61 ^cd^	4.74 ^b^	4.03 ^bc^	1.74 ^cd^	1.70 ^cd^	2.72 ^bcd^	8.14 ^a^	7.77 ^a^	3.10 ^bcd^
